# Supplementary material for: Transposon-modified antigen-specific T lymphocytes for sustained therapeutic protein delivery in vivo
Source: Nat Commun. 2018 Apr 10;9:1325. doi: 10.1038/s41467-018-03787-8 (PMC5893599; doi:10.1038/s41467-018-03787-8)
Supplement: Supplementary file 1 — Supplementary Information [file 41467_2018_3787_MOESM1_ESM.pdf]

Supplementary Information

Transposon-modified antigen-specific T lymphocytes for sustained therapeutic protein delivery *in vivo*

O'Neil et al.

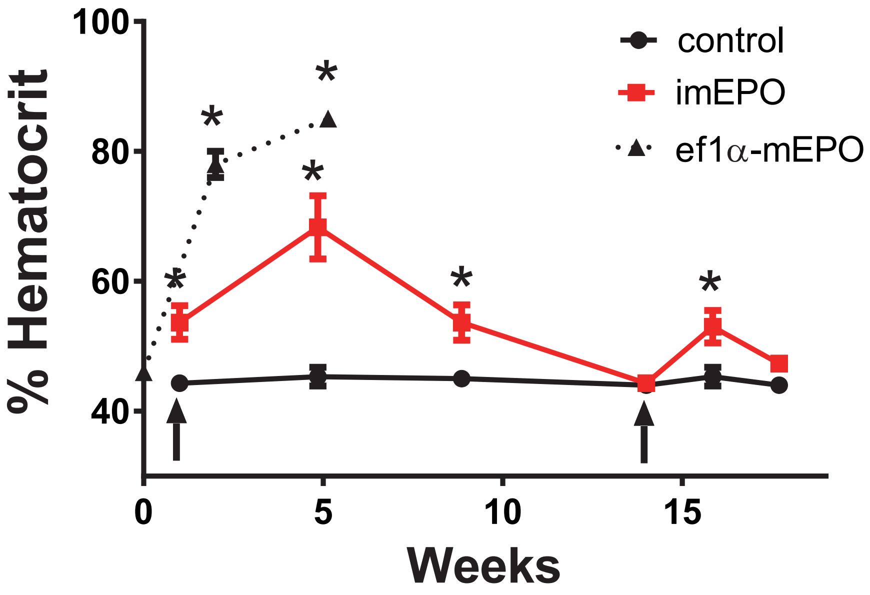

Supplementary Figure 1: Expression of mEPO from mouse liver. Hydrodynamic tail vein injection was used to deliver pCMV-m7pB (5µg) + 25µg pT-EF1α-mEPO (ef1α-mEPO, dark triangles) or pT-Tight-mEPO (imEPO, red squares). Hematocrit was measured at the indicated timepoints. The black arrows indicate with 2mg of doxycycline was given once via intraperitoneal injection. Control animals were untreated. (N=3±SEM; *, $p<0.05$ using student's T test compared to control).

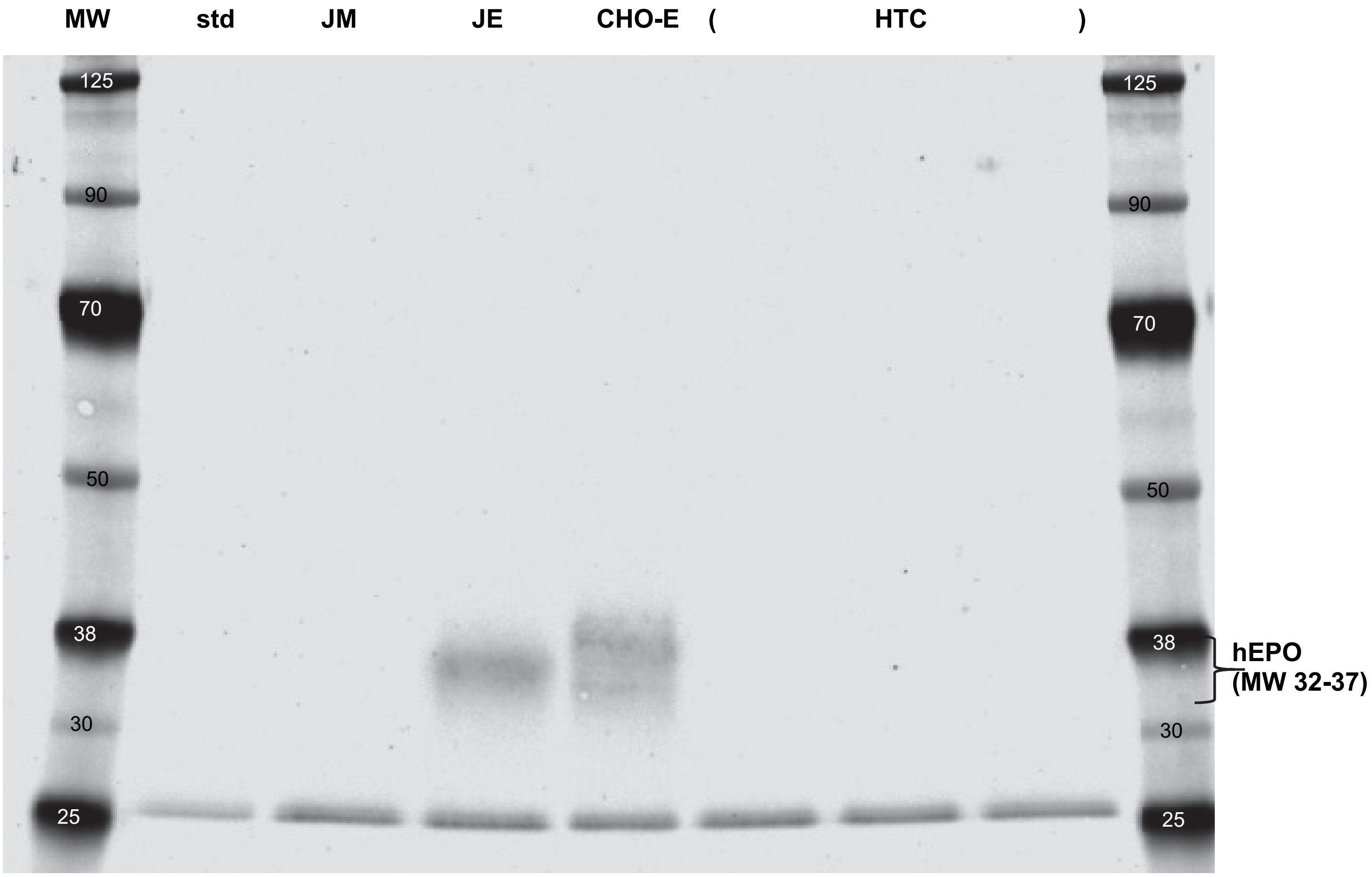

Supplementary Figure 2: Western blot analysis of hEPO expressed from a human T cell line. CHO and human Jurkat T cells were transfected with pT-tight-hEPO and treated with 1mg/ml doxycycline for 24 hours. Media was collected and hEPO was immunoaffinity isolated prior to Western blot analysis for hEPO expression. Std, 84 pg/ml of recombinant hEPO standard from Abcam (not detectable on blot); JM, Jurkat T cell media (control); JE, pT-tight-hEPO transfected and doxycycline treated Jurkat T cells; CHO-E, pT-tight-hEPO transfected and doxycycline treated CHO cells; lanes to the right of CHO-E are unmodified HTC, human T cells. hEPO runs at a molecular weight of 32-37 kDa depending on post-translational modifications. Molecular weight markers are depicted on the left. Shown is a representative of 3 experiments.
